# Framing Political Change: Can a Left Populism Disrupt the Rise of the Reactionary Right? Comment on "Politics, Power, Poverty and Global Health: Systems and Frames"

**DOI:** 10.15171/ijhpm.2017.08

**Published:** 2017-01-17

**Authors:** Ronald Labonté

**Affiliations:** Canada Research Chair, Globalization and Health Equity, Faculty of Medicine, School of Epidemiology, Public Health and Preventive Medicine, University of Ottawa, Ottawa, ON, Canada.

**Keywords:** Global Health Frames, Neoliberal Globalization, Political Populism

## Abstract

Solomon Benatar offers an important critique of the limited frame that sets the boundaries of much of what is referred to as ‘global health.’ In placing his comments within a criticism of increasing poverty (or certainly income and wealth inequalities) and the decline in our environmental commons, he locates our health inequities within the pathology of our present global economy. In that respect it is a companion piece to an editorial I published around the same time. Both Benatar’s and my paralleling arguments take on a new urgency in the wake of the US presidential election. Although not a uniquely American event (the xenophobic right has been making inroads in many parts of the world), the degree of vitriol expressed by the President-elect of the world’s (still) most powerful and militarized country is being used to further legitimate the policies of right-extremist parties in Europe while providing additional justification for the increasingly autocratic politics of leaders (elected or otherwise) in many other of the world’s nations. To challenge right-populism’s rejection of the predatory inequalities that 4 years of (neo)-liberal globalization have created demands strong and sustained left populism built, in part, on the ecocentric frame advocated by Benatar

Somewhat coincidentally, or at least curiously, two people from opposite sides of the world wrote very similar published in this journal scarcely three weeks apart.The two articles do take different trajectories. One, which I wrote,^1^ focused on health promotion, neoliberalism and the Sustainable Development Goals; while the other, by Solomon Benatar,^2^ discussed power, global health, policy/political framing and the environmental crises we now face. But their respective analyses of where health and broader social inequities are located – in a pathological global economy and a supporting hegemonic discourse, defined by me as neoliberal capitalism and by Benatar as individualism and market fundamentalism – are essentially the same.

Benatar’s argument hinged more on an important critique of the limited frame surrounding our recently pumped up interests in ‘global health.’ He argues that global health is not the same as international health, although his characterization of international health as an extension of a ‘charitable…ideologically inspired, individualistic, and biomedical conception’ may be a bit too harsh. There is also a history of a politically critical ‘new internationalism’ that arose around the same time that the post-colonizing ‘developing’ world was pushing for a ‘new international economic order,’ using the bipolar cold war as a leveraging point. While much of what is described as global health today still tends to distil to the patronizing international health Benatar critiques, there have been strong calls to shift such developed/developing (or high-income/low-income) country collaborations into a more critical orientation that interrogates and proposes actions to remediate the inherently global causes and consequences of the high disease burden in poorer countries.

Thus, the West African Ebola outbreak in 2014 was not simply a failure of global health’s limited moral framing, as Benatar posits. Its roots also lay in civil conflicts fueled by rich world exploitation of the countries’ mineral resources; the land-grabbing destruction of its tropical forests that brought fruit bat populations into close human contact^3^; earlier and still current structural adjustment loan conditionalities of the international financial institutions (IFIs) based on neoliberal orthodoxy that weakened public infrastructures (including health systems) and fueled the ‘brain drain’ of already undersupplied health workers to wealthier countries^4^; and years of IFI advice to keep taxes low to attract foreign investment. The result: Sierra Leone, in the two years preceding the Ebola outbreak, gave transnational companies tax holidays worth roughly ten times what it spent annually on its public health programs.^5^ We *know* the inherently global causes (and consequences) of our health inequitable planet.

In arguing for a reconceptualization of what we mean by global health, Benatar usefully describes how our belief systems, frames, cognitive biases and metaphors inform our global health actions. This is particularly urgent given the environmental crises we are now experiencing (his own near-definition of global health is as an ‘ecocentric concept’), and which can no longer be managed through policies or programs embedded in the underlying economic and political forces (and their implicit or explicit values) that construct the delimiting frames within which our global health responses are crafted. He is not alone in taking aim at the Lancet-University of Oslo Commission on global governance for health for its deft diagnoses of our global ills, but exceedingly weak recommendations bound within the norms of existing systems of power and privilege. However, he may be mistaken in attributing this weakness to a ‘lack of moral imagination’ (since the many of the members of the Commission, in my experience of them, have quite vivid moral imaginings), rather than to the very criticism he levels at the Commission: that it exists within hierarchies of power and privilege which make it intrinsically difficult for it to confront those systems of power explicitly in the solutions it proposes. This was also somewhat the case with the World Health Organization’s Commission on Social Determinants of Health,^7^ which was critically strong in its problem analyses but substantially tamer in its recommendations (although these were still punchier than those of the Lancet-Oslo Commission).

Where Benatar and I return to convivial company (and one which I believe most members of the Lancet-Oslo Commission would enjoy) is his conclusion that global health is unlikely to improve without ‘some changes in how the global political economy operates’ (although I would say, more than just *some* changes). I ended my three-week earlier piece in a similar vein, questioning whether we could reform our current and predatory neoliberal capitalism into a gentler, fairer and more sustainable version; or, if not, what visionary system (built, in part, on the ideals implicit in most but not all of the Sustainable Development Goals) might achieve massive popular consensus. What both articles leave only vaguely sketched is how the structures of elite power and privilege might be transformed to diffuse a new moral imaginary (Benatar) or a new global economy (me).

The urgency of such a task is greater now than just a few days ago. I write this commentary just four days after the world’s (still) largest and most militarized economy elected for President a self-proclaiming misogynist, racist, climate-change denying, tax-avoiding (if not evading), narcissistic member of the elite 1% whose wealth derives from exploitative domestic and international business ventures. As pundit analysts have pointed out since, the surprise win for Donald Trump was, at base, the failure of our past 40 years of neoliberal globalization to benefit the majority of people living in high-income countries, and not just those in the United States. Trump is a curious hybrid: an elite anti-elite heaping scorn on the institutions of the liberal bourgeoisie at home and abroad, while using a xenophobic romanticism for a world that never was and a divisive politics of fear and hate that embodies the essence of right-wing populism. There remains little doubt, given similar dynamics playing out in much of Europe, that liberal globalization’s repeated promise that its open markets, hypermobile capital and labour flexibilization will, given enough time, trickle down to benefit all. For most in the older industrialized economies, it has not. For those in the newer industrialized economies who have benefited through outsourced manufacturing, it has come with the social cost of the same upsurge in wealth inequalities that the older, established powers have experienced. For several years the world’s multilateral organizations (even the World Bank and IMF) have cautioned that the lack of equitable sharing in the economic wealth created over the past 40 years would lead to social chaos. This is beginning to happen, and is likely to worsen unless the bases of right-wing populism are challenged, and not just in facts, but in acts. Whether the post-Trump election protests become a sustained change force in the United States remains to be seen; but such protests over a presidential election result (targeting the hate-mongering statements expressed by the candidate during the campaign) may be an American first, at least within living memory.

One wonders what might have transpired in the United States had Bernie Sanders and his middling-left populism become the Democratic candidate. Sanders tapped into the same disenfranchisement experienced by blue collar and largely white men as Trump, and railed equally strongly against the same elites; but he did so from a moderately socialist (more accurately, neo-Keynesian), inclusive and non-divisive platform. European centre-left parties, even when they succeed in gaining power as they have in Greece, have a poor record of being transformative, and without a Sanders-led change in the US Congress and Senate there would have been little chance of his presidency making much of a difference. But at least the analysis and orientation of the electoral protests against the 40 year rule of the 1% might have gone in a different and potentially transformative (rather than reactionary) direction.

And so the critical global health questions for our new era: If there is no longer much popular support across many of the world’s nations for the liberalized globalization that has impoverished and diminished the lives of many, is there a left-populism that could yet avoid the environmental and social cataclysms into which we are now rushing headlong? And, as both Benatar and I hint at, are there sufficiently strong and organized civil society movements at national and global scales to advance a more socialist and ecocentric vision as effectively as our new cohort of right-wing populist demagogues have done with theirs?

Let us hope there is, and work to make it so; for we now face a political and economic climate similar to that which fed World War One, and then World War Two. History may not repeat itself (it cannot), but certain historical patterns can resurrect under similar conditions. We cannot let neo-fascistic right-wing populism (once again) claim the political space of social protest.

## Ethical issues

Not applicable.

## Competing interests

Author declares that he has no competing interests.

## Author’s contribution

RL is the single author of the paper.
